# Voluntary and evoked behavioral correlates in inflammatory pain conditions under different social housing conditions

**DOI:** 10.1097/PR9.0000000000000564

**Published:** 2016-08-09

**Authors:** Claudia Pitzer, Rohini Kuner, Anke Tappe-Theodor

**Affiliations:** a Interdisciplinary Neurobehavioral Core (INBC), University of Heidelberg, Heidelberg, Germany; b Pharmacology Institute, University of Heidelberg, Heidelberg, Germany

**Keywords:** Inflammatory CFA model, Voluntary wheel running activity, CatWalk, Dynamic weight bearing system, LABORAS homecage monitoring, Well-being, Social housing condition

## Abstract

Most preclinical pain models rely on short-duration stimulus-evoked hind paw measurements even though chronic pain is usually a day and night experience. Pain is a debilitating condition that influences the sociability and the ability for voluntary tasks, but the relevant behavioral readouts for these aspects are mostly underrepresented in the literature. Moreover, we lack standardization in most behavioral paradigms. Important aspects are herewith the combination and duration of particular behavioral tasks and the effects of social environment. We aimed at thoroughly investigating stimulus-evoked and voluntary behavioral parameters in the Complete Freund's Adjuvant model of unilateral hind paw inflammation in male mice. Moreover, we analyzed the impact of different social housing conditions. We used a portfolio of classical response measurements, detailed gait analysis, using 2 different measuring systems (Dynamic weight bearing and CatWalk), as well as observer-independent voluntary wheel running and homecage monitoring in a longitudinal time frame. The impact of grouped or isolated housing was investigated in all behavioral paradigms. We observed that unilateral hind paw inflammation provoked changes in several behaviors. Among these were wheel running activity and different homecage activity parameters. Stimulus-evoked hypersensitivity lasted much longer than gait abnormalities and decreased voluntary wheel running activity. Similar effects were monitored in both social housing conditions. This is the first longitudinal study providing detailed insights into various voluntary behavioral parameters related to pain in a unilateral inflammatory model. Stimulus-evoked behavioral changes lasted longer than changes in voluntary behavioral parameters, and the social environment hardly affects these changes.

## 1. Introduction

Classical rodent models of pain are necessary to explore physiological mechanisms and investigate the efficacy of novel analgesics.^19^ Existing models are criticized to not reflect clinical pain characteristics.^6,20,33^ Clinical pain characteristics are mostly of spontaneous nature. Persistent or chronic pain is experienced by day and night. It affects sociability and often the ability for voluntary behavioral tasks. These aspects are severely underinvestigated in rodents and difficult to assess. While patients can describe their pain orally, most rodent studies rely on short-duration stimulus-evoked unilateral hind paw measurements. It is commonly agreed that we need to analyze new parameters that may reflect impairments in the quality of life.^2^ There have been recent attempts to establish nonevoked behavioral measurements to investigate the changes in the animal well-being as a potential readout for the affective component of pain and spontaneous pain. Among these are voluntary wheel running,^6^ homecage monitoring,^33^ dynamic weight bearing (DWB),^6,20,32^ or gait analysis.^35^ These tests are subject of controversy and do not work consistently across laboratories.^30^ There are numerous reasons for this, including the lack of standardization. In addition, most rodent pain studies are performed over short durations during the daytime, when rodents are naturally inactive. Longitudinal measurements, considering behavioral changes in the circadian rhythm, are missing in most studies. Many preclinical studies are therefore limited and cannot represent the full pain picture. Moreover, various other aspects influence behavioral experiments. Among these are the presence and sex of a human observer^29^ and the gender of the animals.^28^ Other factors like over-handling or physical and social enrichment^14,25^ are widely discussed. Applying too many tests or restraining of the animals can lead to stress^19^ and thereby stress-induced analgesia^31^ or stress-induced hyperalgesia.^13^ Furthermore, social isolation harbors stress conditions and can affect the pain behavior.^4,34^

It is time to comprehensively and extensively characterize long-standing models and assess further changes in pain-related daily life well-being.^30^ Especially, longitudinal measurements of voluntary behavior in unrestrained animals are missing and might provide important aspects for better bench-to-bedside translation.

We aimed at thoroughly characterizing the Complete Freund's Adjuvant (CFA) model for inflammatory pain and used a portfolio of classical stimulus-evoked tests and voluntary, observer-independent behavioral tasks to assess pain and pain-related behavioral changes in a preferably observer-independent manner. Among these tests, we performed detailed analyses of static weight and dynamic gait alterations, using 2 different measuring systems. We investigated long-term voluntary wheel running behavior and homecage monitoring to assess the changes in the circadian rhythm. Since animal housing is performed nonuniformly, we analyzed grouped and isolated male mice in this study in all behavioral tests. This is the first study thoroughly characterizing longitudinal voluntary behavioral parameters in CFA mice and includes the impact of social environment.

## 2. Methods

### 2.1. Animals and social housing conditions

C57BL/6N male mice were purchased from Charles River Laboratories (Sulzfeld, Germany) at the age of 8 weeks.

Immediately after delivery, mice were divided into 2 different social housing conditions. For the grouped-housing condition, mice were housed in groups of 3 per cage (named herewith “grouped mice”). Either CFA or control animals were housed together. For the isolated-housing condition, animals were housed individually (named herewith “isolated mice”).

All animals were housed with food and water ad libitum under a standard 12-hour light/dark cycle (light on between 7:00 am and 7:00 pm) with regulated ambient temperature of ±22°C and at relative humidity of 40% to 50%. All procedures were in accordance with the ethical guidelines imposed by the local governing body (Regierungspräsidium Karlsruhe, Germany).

### 2.2. Experimental design and groups

All behavioral experiments started 2 weeks after the arrival of the mice, and behavioral tests were applied up to 9 days after unilateral CFA injection.

Behavioral testing was split in different cohorts because of the large number of behavioral tests and to avoid over-handling of the animals. We investigated 4 cohorts of animals (each 12 animals; 6 mice with persistent pain and 6 control mice) per housing condition. One cohort was analyzed using stimulus-evoked behavioral tests (von Frey test and Hargreaves test) and DWB test. A second cohort was analyzed for their voluntary wheel running behavior, a third cohort was monitored in the LABORAS homecage monitoring system, and another cohort was investigated using the CatWalk system.

**Table:**
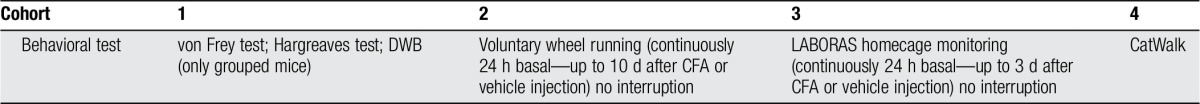

Common animal husbandry and behavioral experiments were performed by females or in an observer-independent manner, to limit experimenter sex-dependent influences.^29^

All behavioral experiments were conducted in a completely randomized and blinded fashion.

### 2.3. Inflammatory pain model

Undiluted CFA (Sigma-Aldrich, Munich, Germany) was injected unilaterally into the intraplantar surface of one hind paw in the mice (20 μL), whereas control mice were injected with 0.9% saline, under isoflurane anesthesia.

### 2.4. Reflexive pain tests

All animals were acclimatized on 4 consecutive days for 1.5 hours to the behavioral setups (von Frey, Hargreaves). von Frey test was performed in the late morning. The Hargreaves test was performed following a 2 hours resting period of the animals.

#### 2.4.1. von Frey test

Mechanical sensitivity was determined using graded von Frey filaments (touch test sensory probes; Stoelting, Dublin, Ireland) with bending forces of 0.07, 0.16, 0.4, 0.6, 1, and 1.4 g on the plantar surface of the hind paw. Filaments were applied with increasing forces, and each filament was tested 5 times with adequate resting periods between each application and the number of withdrawals was recorded. The 40% withdrawal threshold was determined as the von Frey filament (*g* force application), which elicits at least 2 paw withdrawal responses of 5 applications.

#### 2.4.2. Radiant heat pain (Hargreaves test)

The latency of paw withdrawal in response to an infrared beam (which generates a heat ramp) was analyzed using the Hargreaves test (Ugo Basile, Comerio, Italy). Three values per measuring time point were obtained with adequate intermission period between the heat applications.

### 2.5. Nonreflexive behavioral tests

#### 2.5.1. Dynamic weight bearing

We used the DWB system (Bioseb, Boulogne, France) for incapacitance testing in freely moving mice. The system consists of a Plexiglass enclosure (11 × 11 cm) with a floor composed of 1936 pressure transducers. A digital camera was placed at one side of the enclosure. Mice were allowed to move freely within the apparatus for 5 minutes. Pressure data and live video were transmitted via a USB interface to a PC containing DWB software version 1.3. After the completion of the test, mice were removed and the test chamber was cleaned with alcohol wipes. For data analysis, the raw pressure data were automatically synchronized with time-lapse video images. Each test segment was manually validated ensuring that each weight zone corresponded to the appropriate assigned paw. The system enabled the analysis of the paw weight distribution and the paw print area. Animals were acclimatized for 2 sessions before basal measurement.

#### 2.5.2. “CatWalk”-based analysis

The CatWalk XT version 10.6 gait analysis system (Noldus Information Technology, Wagening, the Netherlands) consists of an enclosed 1.3 m black corridor on a glass plate, which is illuminated inside with a green LED. This light is internally reflected, except at those areas where the animal makes contact with the glass plate. Wherever the paws touch the glass, light is refracted on the opposite side. Using the Illuminated footprints technology, paws are captured by a high-speed video camera that is positioned underneath the glass. The mouse is placed on one end of the corridor and allowed to transverse it voluntarily. The brightness of a pixel depends on the amount of light received from a paw area by the camera. The system enables an automatic footprint classification, error correction, interactive footprint measurements, and data segmentation profiling.

We used the following parameters:

(1) Paw print area represents the surface of the complete print of a paw.
(2) Maximal contact intensity of a paw. The intensity of a print that depends on the degree of contact between a paw and the glass plate. This parameter increases with increasing weight. Therefore, intensity is a measure of weight placed on the glass plate.
(3) Swing phase is the duration of no contact of a paw with the glass plate in a step cycle.
(4) Stride length is the distance between successive placements of the same paw.
(5) Stand is the duration of ground contact for a single paw.
(6) Duty cycle expresses the stand as a percentage of a step cycle (step cycle is the time between 2 consecutive initial contacts of the same paw. Step cycle = stand + swing). Duty cycle = stand/(stand + swing) × 100%.

Mice were habituated to the CatWalk setup and allowed to cross the corridor for 3 sessions. On each testing day, animals were allowed to cross the corridor 3 times.

Gait analysis was performed until measuring parameters reached basal levels.

#### 2.5.3. Homecage monitoring

The LABORAS (Laboratory Animal Behaviour Observation, Registration, and Analysis System) homecage observation (Metris b.v., Hoofddorp, the Netherlands) is a system that uses a carbon fiber platform to detect behavior-specific vibration patterns produced by the animal. A homecage is placed on top of the platform and the specific LABORAS software version 2.6 processes the produced vibrations into various behavioral parameters. Continuously, we analyzed climbing, grooming, rearing, locomotion, and immobility. These behavioral parameters were calculated over time or as frequency counts. Additional tracking information like travelling distance, average or maximal speed was collected. Animals were placed individually in the calibrated cage under standard housing condition with free access to food and water at all measuring days in the morning, usually around 8 am. We continuously monitored homecage activity over 24 hours before mice were taken out of the cage for CFA or saline injection. Directly after intraplantar injection, mice were placed back into the same cage and monitored continuously for 72 hours without any interruption or disturbance.

#### 2.5.4. Voluntary wheel running activity

Animals were placed individually in cages containing a running wheel and free access to food and water. Unrestricted voluntary wheel running activity was digitally recorded using the AWM (activity wheel monitoring) counter (Lafayette Instrument Company, Lafayette, IN), which uses an optical sensor to detect the total revolutions of the wheel and is connected to a USB interface and PC running an AWM software (Lafayette Instrument). We continuously monitored voluntary wheel running activity over 24 hours before mice were taken out of the cage for CFA or saline injection. Directly after intraplantar injection, mice were placed back into the same cage and monitored continuously for 10 days without any interruption or disturbance.

### 2.6. Statistical analysis

For all measurements, data were calculated and presented as mean ± SEM. Unless stated otherwise, 2-way repeated-measures analysis of variance followed by post hoc Tukey tests was used to assess statistical significance. Changes with *P* <0.05 were considered to be significant.

## 3. Results

### 3.1. Stimulus-evoked behavior and body weight

**Figure 1.**
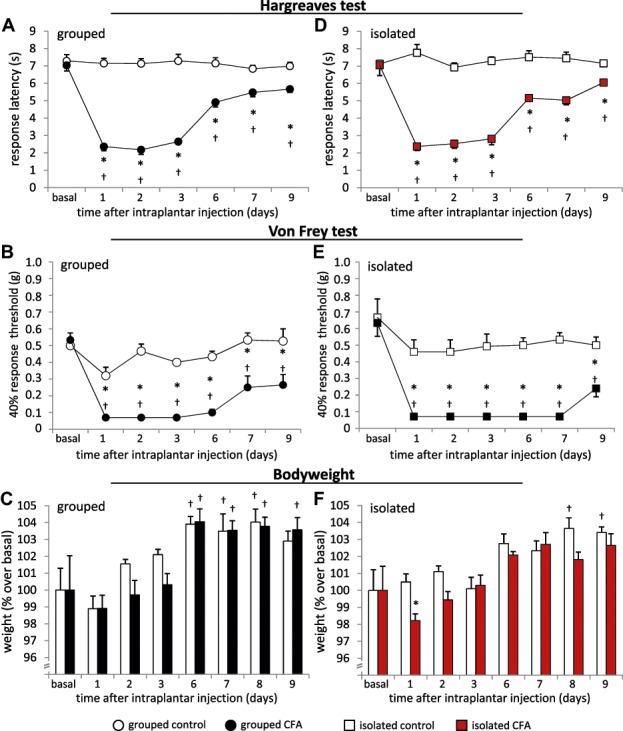
Analysis of nociceptive sensitivity and body weight following unilateral paw inflammation induced by Complete Freund's adjuvant (CFA). All left column panels (A-C) show results from animals which were housed in groups (black circular symbols or black bars), all right column panels (D-F) show results from CFA and control animals which were housed individually (red square symbols or red bars). (A, D) Time course of withdrawal latency to radiant heat, (B, E) 40% response threshold towards the application of graded von Frey hair filaments, and (C, F) analysis of body weight changes over basal body weight up to 9 days following CFA or vehicle injection. N = 6 mice/group, *P* < 0.05 indicated by “*” as compared with control group, “†” as compared with basal values within a group, 2-way repeated-measures analysis of variance with post hoc Tukey test. All data points represent mean ± SEM.

Complete Freund's Adjuvant–injected animals developed thermal hyperalgesia and mechanical allodynia at the injected paw (Figs. 1A and B). They showed a significant decrease in paw withdrawal latency in response to an infrared heat stimulation using the Hargreaves test (Fig. 1A) and a significant increase in response frequency to mechanical punctate (von Frey) stimuli (Fig. 1B). Changes in withdrawal response peaked during the first 3 days and remained significant over the whole observation period of 9 days after CFA injection (Figs. 1A and B). In addition, we measured the body weight of all mice. There was no change in body weight over the first 3 days after CFA injection. Complete Freund's Adjuvant and control mice showed a significant increase in body weight from day 6 on as compared with their basal body weight (Fig. 1C).

We went on to investigate stimulus-evoked hind paw measurements in CFA and control mice which were housed individually. The injection of CFA led to a significant decrease in paw withdrawal latency in response to increased infrared heat over the whole observation period (Fig. 1D) and an increase in response frequency towards mechanical stimuli (Fig. 1E). With respect to the body weight, we found a significant reduction at 24 hours after CFA injection as compared to the control mice (Fig. 1F). Isolated control animals gained significant weight from day 8 after vehicle injection, whereas isolated CFA mice did not show any significant gain in body weight during the observation period (Fig. 1F).

The magnitude and time course of thermal hyperalgesia and mechanical allodynia was similar between grouped and isolated animals after CFA injection.

### 3.2. Weight distribution and gait analysis

We were interested to investigate various gait parameters and thereby compare 2 different measuring systems: the DWB system (Bioseb) and the CatWalk system (Noldus). Gait analysis was performed until measuring parameters reached basal levels. All measuring parameters were illustrated as ratio of the inflamed (left) hind paw (LH) over the noninflamed (right) hind paw (RH).

The DWB system (Bioseb) enables the investigation of static weight parameters as paw weight distribution and paw print area, in freely moving mice, whereas the CatWalk system enables complete automatic gait analysis of these static parameters as well as dynamic gait parameters such as stride length, stand duration, and swing phase, among others.

**Figure 2.**
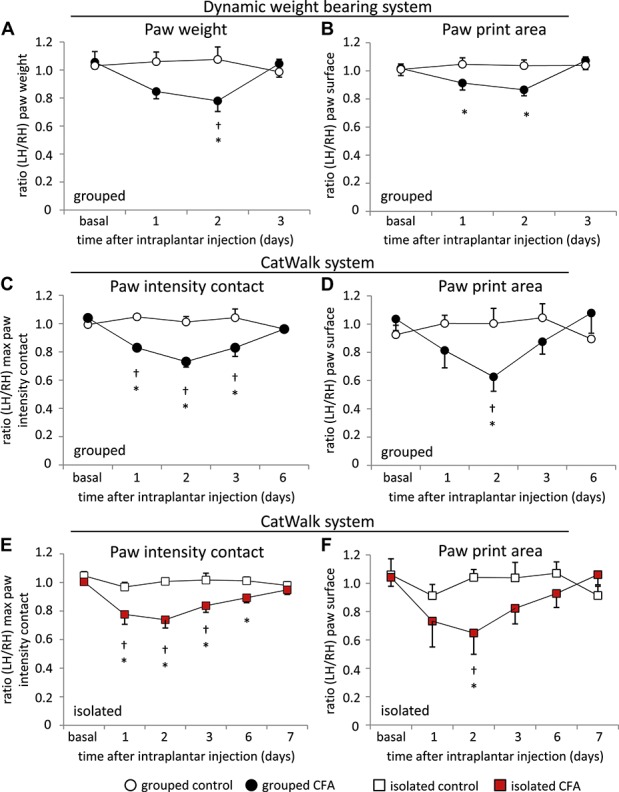
Changes in static weight bearing following Complete Freund's adjuvant (CFA) unilateral hind paw inflammation in animals which were housed in groups (black circular symbols) (A-D) and animals which were housed individually (red square symbols) (E, F). Grouped animals were analyzed using the Dynamic weight bearing system (Bioseb) (A, B) and the CatWalk system (Noldus) (C-F). The ratio of the left over the right hind paw is shown for (A) paw weight, (B) paw print area, (C, E) paw intensity contact, and (D, F) paw print area. N = 6 mice/group, *P* < 0.05 indicated by “*” as compared with control group, “†” as compared with basal values within a group, 2-way repeated-measures analysis of variance with post hoc Tukey test. All data points represent mean ± SEM.

Using the DWB system, we saw that CFA-injected mice put less weight on the inflamed hind paw over the noninflamed paw at day 1. This observation was significantly more pronounced at day 2 after CFA injection (Fig. 2A). At 3 days after CFA injection, mice showed again an equal weight distribution of both hind paws (Fig. 2A). Similarly, we measured a significant decrease in paw surface area over the first 2 days after CFA injection (Fig. 2B).

Furthermore, we used the CatWalk system and analyzed comparable static gait parameters. We found a significant decrease in the paw intensity contact over 3 days after CFA injection (Fig. 2C). At day 6, no difference in paw intensity contact was detectable anymore (Fig. 2C). In addition, the paw print area dropped after CFA injection and was maximally and significantly pronounced on day 2 (Fig. 2D). This decrease diminished on day 3 (Fig. 2D).

Because of the high degree of comparability between the results of the grouped mice with the DWB system and the CatWalk system, we solely analyzed isolated CFA animals with the CatWalk apparatus.

Isolated CFA mice showed a significantly decreased paw intensity contact during the first 3 days (Fig. 1E) and a significant drop in paw print area on day 2 after CFA injection (Fig. 2F).

**Figure 3.**
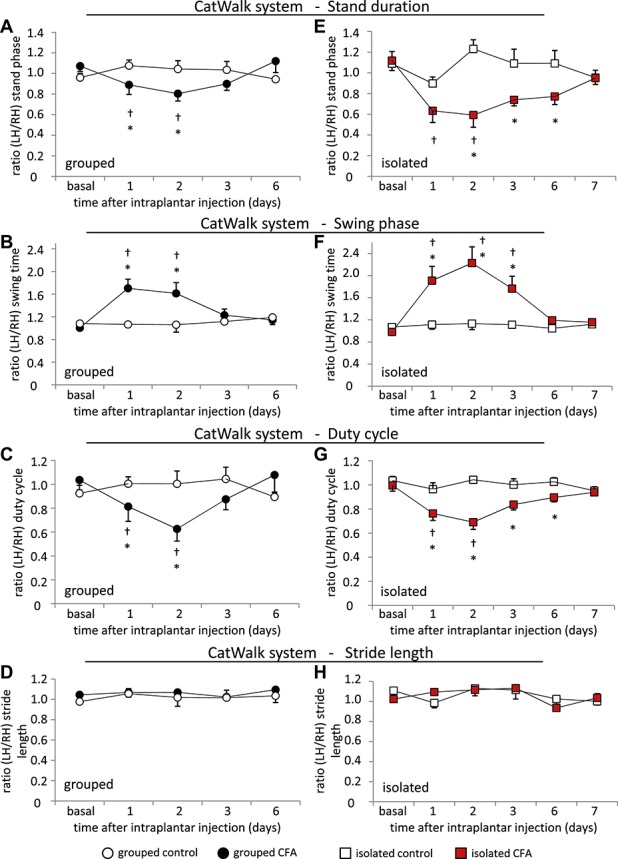
Changes in dynamic weight bearing following CFA unilateral hind paw inflammation in animals which were housed in groups (black circular symbols) (A-D) and animals which were housed individually (red square symbols) (E-H). The ratio of the left over the right hind paw is shown for (A, E) stand duration, (B, F) swing phase, (C, G) duty cycle, and (D, F) stride length. N = 6 mice per group, *P* < 0.05 indicated by “*” as compared with control group, “†” as compared with basal values within a group, 2-way repeated-measures analysis of variance with post hoc Tukey test. All data points represent mean ± SEM.

We further analyzed more dynamic gait parameters using the CatWalk system. There was a significant drop in the stand duration (Fig. 3A) and a significantly increased swing time (Fig. 3B) of the ipsilateral paw during the first 2 days following inflammation in CFA-grouped animals. In addition, the duty cycle was significantly reduced on days 1 and 2 following inflammation (Fig. 3C). We did not find any change in the stride length between CFA and control mice over the whole observation period (Fig. 3D).

We went on and analyzed individually housed CFA and control animals (Figs. 3E–H). Isolated CFA mice showed a decreased stand duration of the inflamed hind paw, which peaked at day 2 after CFA injection and slowly diminished until day 7 (Fig. 3E). The swing duration increased significantly over 3 days in CFA mice (Fig. 3F), and we observed a significant decrease in the duty cycle, which was maximally pronounced during the first 2 days after CFA injection (Fig. 3G). No change in stride length was measurable between CFA mice and control mice of the isolated cohorts (Fig. 3H).

Grouped and isolated CFA mice showed significant changes in static as well as dynamic gait parameters, which were significantly altered during the first 2 to 3 days after CFA injection. Grouped CFA mice showed normal gait characteristics at 6 days after CFA injections, whereas gait parameters reached basal levels after 7 days in isolated mice (Figs. 2C–F, 3).

### 3.3. Voluntary wheel running

**Figure 4.**
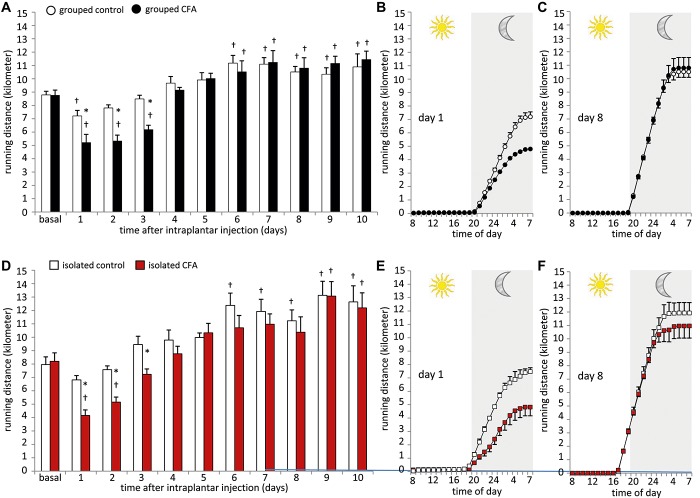
Effect of Complete Freund's adjuvant (CFA)-induced inflammation on voluntary wheel running behavior. All mice were analyzed continuously for 24-hour basal running and up to 10 days after 20 μL CFA or vehicle unilateral hind paw injection without any interruption. (A) Running distance (in kilometers) of CFA and control mice, which were housed in groups until separation and start of voluntary wheel running analysis. (B, C) Typical running profiles of previously grouped mice at (B) day 1 and (C) day 8 after CFA or vehicle injection. (D) Running distance (in kilometers) of CFA and control mice which were housed individually before the start of voluntary wheel running analysis and typical running profiles of these isolated cohorts at (E) day 1 and (F) day 8 after paw injection of CFA or vehicle. N = 6 mice per group, *P* < 0.05 indicated by “*” as compared with control group, “†” as compared with basal values within a group, 2-way repeated-measures analysis of variance with post hoc Tukey test. All data points represent mean ± SEM.

So far, a longitudinal investigation of voluntary wheel running behavior following unilateral hind paw inflammation has not been reported in mice. To properly assess phase-dependent changes in voluntary wheel running activity, we continuously monitored wheel running activity for 24 hours before and immediately after, without any interruption for 10 days following unilateral CFA hind paw inflammation (Fig. 4). We first studied mice which were initially housed in groups and had to be separated for the wheel running experiment. Control mice showed a significant drop in running distance during the first 24 hours (day 1) after 20 μL injection of 0.9% NaCl (Fig. 4A). This difference diminished on day 2, and the daily running distance of control mice increased constantly until day 6, from which on animals showed a stable running behavior until the end of the observation period (Fig. 4A). Interestingly, mice after unilateral CFA hind paw injection used the wheel significantly less during the first 3 days as compared with their basal running behavior and as compared with control mice (Fig. 4A). From day 4 on, there was no difference in wheel running activity visible between CFA and control mice. As seen in control mice, CFA animals showed a significantly increased and stable wheel running profile from day 6 on, until the end of the observation period (Fig. 4A).

The continuous measurement of voluntary wheel running behavior allowed us to assess activity changes between illuminated (day) and dark (night) periods. All mice showed a classical night activity profile in voluntary wheel running behavior (Figs. 4B and C). They used the wheel mainly between 9:00 pm and 4:00 am, whereas hardly any running was performed during the daytime (Figs. 4B and C). Typical representative running profiles of CFA- or vehicle-injected mice are shown for day 1 in Figure 4B and for day 8 in Figure 4C.

We performed the same experiments with mice which were housed individually already before the start of the experiment (isolated mice). After vehicle injection, the daily running distance of control animals dropped slightly over 2 days (Fig. 4D) and increased continuously until animals reached a stable running profile from day 6 on (Fig. 4D). Unilateral hind paw inflammation led to a significant decrease in voluntary wheel running over the first 2 days as compared with the basal running distance and over the first 3 days as compared with control mice (Fig. 4D). We measured a continuous increase in daily running distance of CFA mice, which became significantly increased over their basal running behavior on days 9 and 10 (Fig. 4D). As seen with the grouped cohort, isolated mice showed a classical night activity profile in voluntary wheel running behavior (Figs. 4E and F). The running profile of day 1 is shown in Figure 4E and day 8 in Figure 4F.

There were no major differences in voluntary wheel running behavior between previously grouped animals and animals which were kept individually before the start of the experiment. In both experimental conditions, the CFA injection led to a significant reduction in voluntary wheel running activity during the first 2 to 3 days after unilateral hind paw injection, as compared with their basal running behavior and as compared to the control animals at the same time point.

### 3.4. Homecage monitoring

**Figure 5.**
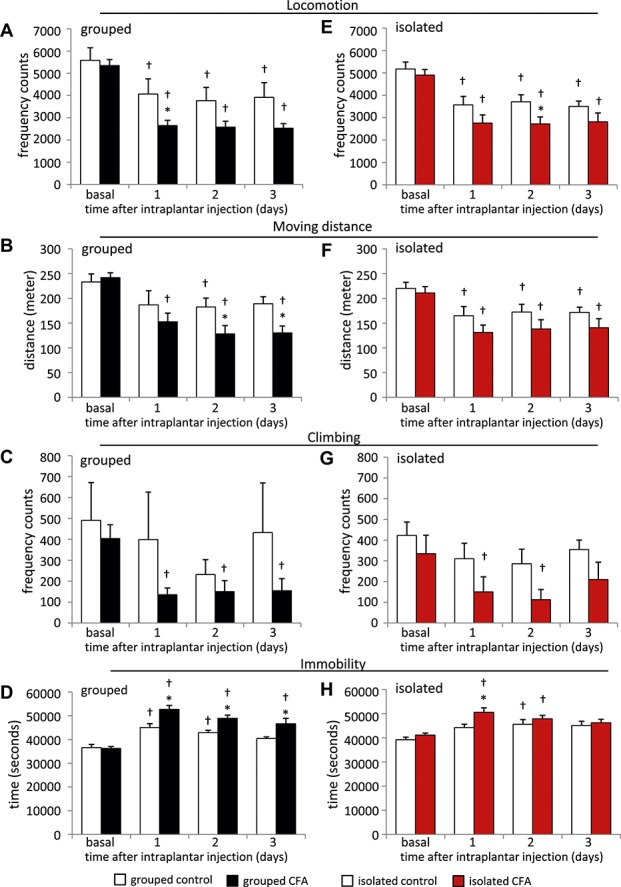
Homecage behavior of mice following unilateral Complete Freund's adjuvant (CFA) inflammation. Animals were analyzed continuously for 24-hour basal measurement and up to 3 days after 20 μL CFA or vehicle unilateral hind paw injection. All left column panels (A-D) show results from CFA and control animals which were previously housed in groups (black bars), and all right column panels (E-H) show results from CFA and control animals which were housed individually (red bars). Data per analysis time point represent a 24-hour measuring time period. (A, E) Locomotion frequency, (B, F) total moving distance (meters), (C, G) climbing frequency counts, and (D, H) total immobility time (seconds). N = 7 control-grouped mice, 8 CFA-grouped mice, and 6 mice per group for isolated animals, *P* < 0.05 indicated by “*” as compared with control group, “†” as compared with basal values within a group, 2-way repeated-measures analysis of variance with post hoc Tukey test. All data points represent mean ± SEM.

**Figure 6.**
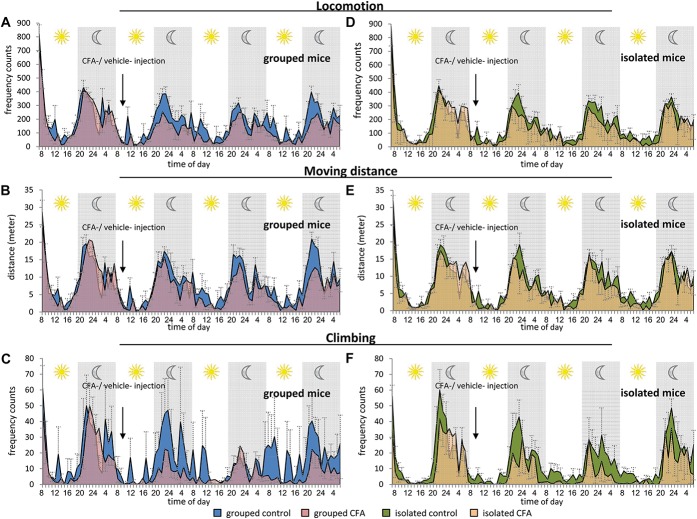
Time profile of homecage behavior of mice following unilateral CFA inflammation. Animals were analyzed continuously for 24-hour basal measurement and up to 3 days following 20 μL CFA or vehicle unilateral hind paw injection. All left column panels (A-C) show results from CFA and control animals which were previously housed in groups, and all right column panels (D-F) show results from CFA and control animals which were housed individually. Blue background curves represent data from control animals, and red curves represent behavioral data from CFA animals. (A, D) Locomotion frequency, (B, E) total moving distance (in meters), and (C, F) climbing frequency counts. N = 7 control-grouped mice, 8 CFA-grouped mice, and 6 mice per group for isolated animals. All data points represent mean ± SEM.

To assess innate behavioral parameters, we performed homecage monitoring analyses using the automated LABORAS system. We started the measurements with grouped animals, which had to be separated into individual cages. Following basal behavioral analysis over 24 hours, CFA or vehicle was injected unilaterally in the plantar surface of the hind paw. Animals were placed back into the LABORAS cages and monitored continuously for another 3 days without any disturbance (Figs. 5A–D, 6, Table 1). Generally, behavioral activity to a novel environment results in increased exploration, which is reflected in an increase of moving distance, moving speed, locomotion, climbing, and rearing during the first hours (Fig. 6). In line with this, we observed a drop in various behavioral parameters in control mice following basal measurements (Fig. 5, Table 1). We measured a drop in locomotion frequency (Fig. 5A), moving distance (Fig. 5B), locomotion duration, average moving speed and rearing frequency (Table 1) in control mice following basal exploration. The climbing frequency of control animals did not change significantly (Fig. 5C), but control animals showed an increased duration of immobility on days 1 and 2 after vehicle injection compared with their basal behavior (Fig. 5D). Interestingly, over and above these behavioral changes which were caused by increased environmental exploration, we found significant alterations of various behavioral parameters in CFA-injected animals (Figs. 5A–D, 6A–C). Complete Freund's Adjuvant animals showed a significantly decreased locomotion frequency during the first day after CFA injection (Fig. 5A). They moved significantly less on day 1 after CFA injection compared with their basal moving distance, but also significantly less on days 2 and 3 compared with control animals (Fig. 5B). In addition, CFA mice showed significantly reduced average moving speed, locomotion duration (Table 1), or climbing frequency (Fig. 5C) and an increased immobility duration throughout the whole observation period (Fig. 5D). These alterations were more pronounced during the dark night phase than during the bright day phase (Figs. 6A–C).

**Table 1:**
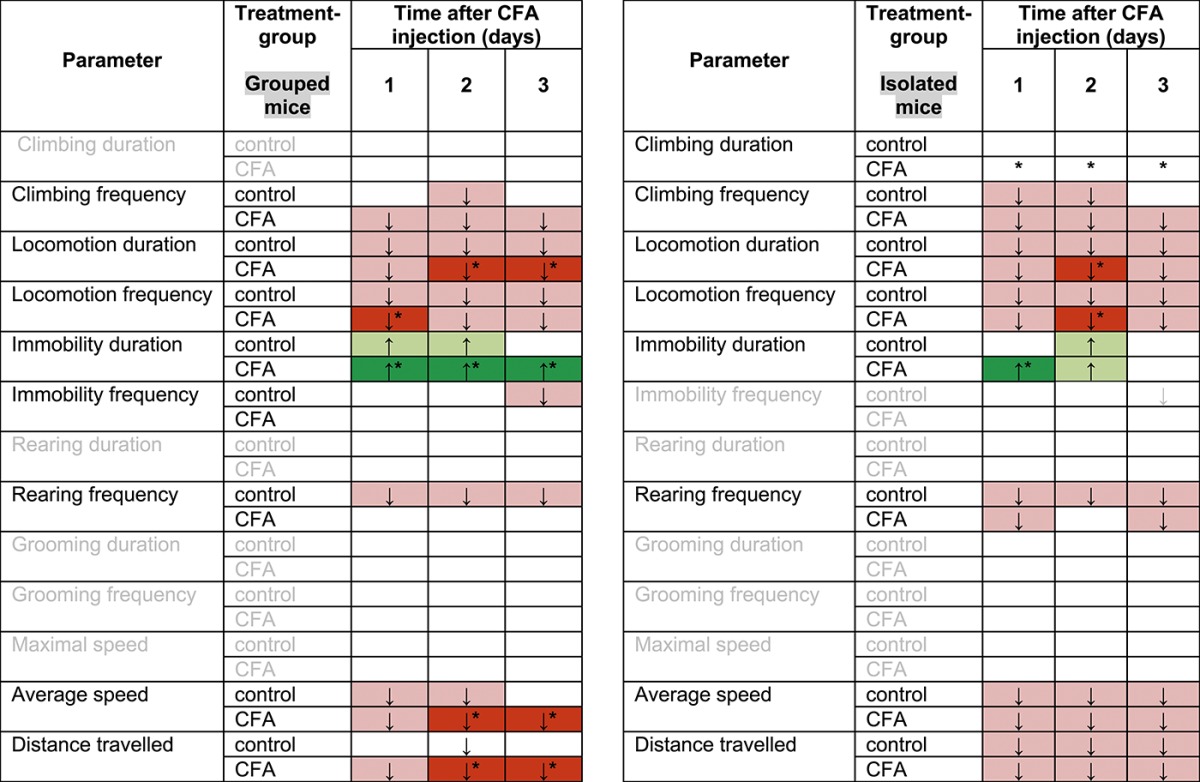
Homecage behavior of mice after unilateral Complete Freund's Adjuvant (CFA) inflammation.

As with all other behavioral tests, we examined mice which were single housed (isolated) before the start of the LABORAS homecage monitoring. After 24-hour basal exploratory measurements, we observed similar behavioral alterations as seen with the grouped cohorts for most behavioral parameters (Figs. 5E–H, 6, Table 1). All mice showed a drop in locomotion frequency following basal measurements (Fig. 5E), which was more pronounced in CFA animals and significantly different to control animals on day 2 after CFA injection (Fig. 5E). We measured a significant drop in moving distance of CFA and control mice after basal measurement and unilateral paw injection (Fig. 5F), and CFA mice showed a trend towards less movement as compared with control mice, but this was not significant (Fig. 5F). Complete Freund's Adjuvant injection led to a significant decrease in climbing frequency during the first 2 days after injection, whereas the climbing frequency was unchanged in control animals (Fig. 5G). In addition, CFA mice showed significantly increased immobility duration on day 1 (Fig. 5H). The locomotion duration, average moving speed, and rearing frequency was significantly reduced in control and CFA animals but not significantly altered between CFA and control mice (Table 1). Behavioral differences were more pronounced during the night period than during the daytime (Figs. 6D–F).

We observed various alterations in homecage activity between CFA and control animals. Both experimental groups of CFA animals (grouped and isolated) showed significantly increased immobility behavior, reduced locomotion, and moving behavior as compared with control mice.

### 3.5. General observations

We did not aim to directly compare grouped vs isolated male animals. This would also not be possible with respect to the longitudinal measurements requiring permanent isolation in the wheel running cages and LABORAS cages. Nevertheless, there seem to be some differences in behavioral results that might be related to the social environmental conditions.

It seems that isolated CFA mice showed a more pronounced response to mechanical stimuli (Fig. 1E) as do grouped CFA mice (Fig. 1B). Only isolated CFA mice show a reduced body weight at day 1 after CFA injection, and all isolated mice gained less weight than mice which were housed in groups (Figs. 1E and F). Differences in diverse gait parameters lasted longer in isolated mice than in grouped animals (Figs. 2C–F, 3). Furthermore, it appears that the observed behavioral changes in the LABORAS homecage monitoring experiment were more pronounced in mice which were previously grouped (Figs. 5 and 6, Table 1). Since the emphasis of this study is not on the effect of social isolation, behavioral experiments of grouped and isolated cohorts were not performed in parallel; hence, we would be careful with further detailed speculations about the reasons.

## 4. Discussion

This is the first study that thoroughly investigating the inflammatory CFA mouse model for voluntary long-term behavioral changes, including circadian rhythm and the effect of social housing conditions in male animals. We used a portfolio of diverse behavioral paradigms, and thus far, none of the tests has been applied longitudinally and there is also no publication available, directly comparing the DWB system and the CatWalk system in the CFA model. The results of the different behavioral paradigms are discussed below in details.

### 4.1. Stimulus-evoked behaviors and body weight

Irrespective of the social housing conditions, all CFA animals developed significant mechanical and thermal hyperalgesia. Interestingly, we found a significant initial weight loss in isolated CFA mice compared with control mice, whereas we did not observe any difference between grouped CFA and grouped control mice. A loss in body weight has been shown in different animal pain models,^3,5,18,27,33^ but information on social housing conditions is not always provided and causes are not elucidated. It is very likely that isolated CFA mice have a reduced food and water consumption on the first day based on impaired well-being, which is less pronounced in social enriched CFA mice. One previous report compared the body weight systematically between isolated and grouped mice,^25^ and consistent with our findings, Pham et al. reported that grouped mice gained more weight after surgical intervention than isolated mice.^25^

### 4.2. Gait analysis

There have been several attempts to analyze gait changes in chronic or persistent pain models, but the value of this investigation is controversially discussed. Most people argue that gait alterations are related to pain,^1,7,23,26^ whereas others do not.^20^ There is evidence that gait changes in inflammatory models are based upon inflammatory pain,^26^ whereas they result from motor system perturbations in neuropathic pain models.^26^ Many factors including differences in the rodent strain, the pain model, the investigation time point, and the measuring system might influence the results.

We thoroughly investigated gait alterations in CFA animals using 2 different systems, the DWB system (Bioseb) and the CatWalk system (Noldus). This detailed and comparative attempt has never been performed before and revealed stable and reproducible results. Consistent with previous reports, we observed that intraplantar CFA injection leads to diminished paw pressure and print area of the inflamed paw.^6,12^ Dynamic gait parameters have not been investigated in this context before. In general, we observed that unilateral CFA hind paw inflammation led to significant alterations in various static and dynamic gait parameters. These changes were maximally pronounced within the first 2 to 3 days after CFA injection and diminished within 6 or 7 days in the grouped or isolated cohort, whereas stimulus-evoked hyperalgesia and allodynia were still significantly pronounced. It is possible that the observed limping behavior is elicited from increased contact sensitivity, pain avoidance behavior, or spontaneous pain.^6^ Successful measures of spontaneous pain using the conditioned place preference (CPP) test have only been shown in the initial phase of the CFA model (reviewed in Refs. 21,30). In addition, spontaneous foot lifting has only been observed during the first 1 to 2 days after CFA injection, based upon ectopic c-fiber activity.^8^ It is therefore likely that the temporary gait irregularities in the initial phase of the CFA model result from additional spontaneous pain. We would recommend the detailed gait analysis as a meaningful tool to analyze pain-related behavioral changes in the CFA model.

### 4.3. Voluntary wheel running

Voluntary wheel running has been proposed as an observer-independent measure for ongoing pain in inflammatory models.^6,9,15^ Previously reported significant differences between CFA and control animals have been assessed using bilateral CFA injection and using a 1-hour wheel running protocol.^6,9^ We performed long-term measurements over 10 days following unilateral paw inflammation, including circadian analyses to comprehensively assess an innate voluntary wheel running activity. Consistent with other studies, we measured a stable and constant wheel running activity in grouped control mice after 6 days of wheel usage.^24^

Unilateral CFA inflammation led to a significant reduction in voluntary wheel running activity during the first 2 to 3 days in both grouped and isolated animals. Vehicle-injected mice showed a reduced wheel running activity on the injection day. This could originate from either reduced exploration of the running wheel or from pain caused by vehicle injection.

It is possible that our long-term measurement unmasked previously uncovered significant changes in wheel running activity, where bilateral CFA injection was necessary to assess significant changes in a 1-hour recording period in mice or rats.^6,9^ The decreased wheel running behavior of CFA mice lasted shorter than mechanical and heat hypersensitivity. This finding is consistent with previous reports^6,9^ and might also be an indication of spontaneous pain during the initial phase, as described above, rather than avoiding painful episodes via movement and physical activity. Interestingly, we did not observe any tremendous difference between mice which were housed in groups or individually before the measurements. Importantly, our data clearly show the value of measurements including the circadian rhythm. From our results, it appears that voluntary wheel running is an effective measure to examine significant voluntary behavioral changes associated with unilateral hind paw inflammation in mice and that this behavior is not affected by social isolation during the measuring period.

### 4.4. Homecage monitoring

We used homecage monitoring to assess objective, observer-independent behavioral parameters in a familiar cage environment. Continuous monitoring of CFA and control mice revealed significant changes following basal measurement, which were most likely caused by reduced novel cage exploration. Nevertheless, a variety of behavioral parameters were significantly different between CFA and control mice, and these changes were more pronounced during the night phase. Among these were reduced locomotion and increased immobility. Recently, Urban et al.^33^ reported no impairment of basic daily life activity parameters like moving distance in CFA mice. In this context, it is important to consider that Urban et al. used a different homecage monitoring system and injected 10 μL of CFA twice, with a 1-week interval, compared to a single 20 μL injection which we used in this study.

It is noteworthy that behavioral alterations were more pronounced in mice which were already housed in groups before the homecage monitoring than in initially isolated mice. Social isolation harbors stress conditions, and recently, it has been shown that stress aggravates chronic pain in rodents,^16^ and this might contribute to the slightly more pronounced behavioral alterations in previously grouped mice.

We recommend the analysis of innate behavioral parameters in a homecage system as a valuable tool to assess inflammation-induced behavioral changes in general well-being.

### 4.5. General observations based upon different social housing conditions

It seems that social housing conditions have an impact on the behavioral results in the CFA model. Isolated CFA mice seem to have an increased stimulus-evoked response behavior, and they gain less weight and show a more pronounced gait phenotype. Behavioral parameters in the homecage analysis seem to be more pronounced in grouped mice than in isolated mice. It has been shown that social isolated mice show reduced mechanical allodynia in the CFA model as well as in a neuropathic mouse model.^11^ Interestingly, others showed a significantly increased mechanical allodynia in isolated neuropathic pain mice.^22^ These differences might arise from different mouse strains, isolation periods, and testing protocols.

### 4.6. Conclusion and outlook

It has been discussed that we need to develop better animal pain models mimicking human pain conditions.^33^ To step along that direction, there is an urgent need to expand the portfolio of behavioral measurements beyond the classical stimulus-evoked tests to better understand the existing preclinical pain models before we start to develop novel models. This will bring us closer to a better bench-to-bedside translation. Moreover, given the importance to study sex differences in pain^10^ and growing literature on potential underlying mechanisms,^17,28^ it would be interesting to study the impact of social isolation also in female mice. In this study, we focused on detailed behavioral characterization of different voluntary paradigms in C57BL6/N male mice, under different commonly practiced social housing conditions. Future studies directly addressing the impact of isolation would be interesting.

We could show that the CFA model implicates alteration in static and dynamic gait parameters, wheel running, and homecage activity. Voluntary behavioral changes occurred in a time-dependent manner which lasts shorter than stimulus-evoked hypersensitivity.

From the above described data, it appears that different social housing conditions have only a minor influence on the motivation for voluntary tasks in the CFA model.

## Conflict of interest statement

The authors have no conflicts of interest to declare.

This work was supported by grants from the Deutsche Forschungsgemeinschaft to A. Tappe-Theodor (individual grant and SFB 1158 Project S01).
